# ﻿A global analysis of the effectiveness of policy responses to COVID-19

**DOI:** 10.1038/s41598-023-31709-2

**Published:** 2023-04-06

**Authors:** Kwadwo Agyapon-Ntra, Patrick E. McSharry

**Affiliations:** 1 Carnegie Mellon University Africa, Kigali, Rwanda; 2 African Centre of Excellence in Data Science, University of Rwanda, Kigali, Rwanda; 3 Oxford MAN Institute of Quantitative Finance, University of Oxford, Oxford, UK

**Keywords:** Health policy, Infectious diseases, Viral infection, Respiratory tract diseases, Psychology and behaviour, Statistics

## Abstract

Governments implemented many non-pharmaceutical interventions (NPIs) to suppress the spread of COVID-19 with varying results. In this paper, country-level daily time series from Our World in Data facilitates a global analysis of the propagation of the virus, policy responses and human mobility patterns. High death counts and mortality ratios influence policy compliance levels. Evidence of long-term fatigue was found with compliance dropping from over 85% in the first half of 2020 to less than 40% at the start of 2021, driven by factors such as economic necessity and optimism coinciding with vaccine effectiveness. NPIs ranged from facial coverings to restrictions on mobility, and these are compared using an empirical assessment of their impact on the growth rate of case numbers. Masks are the most cost-effective NPI currently available, delivering four times more impact than school closures, and approximately double that of other mobility restrictions. Gathering restrictions were the second most effective. International travel controls and public information campaigns had negligible effects. Literacy rates and income support played key roles in maintaining compliance. A 10% increase in literacy rate was associated with a 3.2% increase in compliance, while income support of greater than half of previous earnings increased compliance by 4.8%.

## Introduction

### Background

COVID-19 is generally believed to have originated in Wuhan, China in late 2019^1^. By the 11th of March 2020, the virus had been detected in 114 countries across the world and was officially declared a pandemic by the World Health Organization (WHO)^2^. Government responses to the pandemic have varied dramatically with time^3,4^. For federal governments like that of the United States of America, these variations in responses are present even at the state level^5^. Following the declaration of a pandemic by WHO, there were widespread global lockdowns in March of 2020. More disparate measures were implemented in countries across the globe over the following months. These varying policy responses produced different outcomes not just in terms of how the virus spread, but also with regard to how different individuals reacted to restrictions, adverse effects on national economies^6^, and decreasing the personal well-being of billions of people across the planet, especially with respect to the declining mental health of people during lockdowns^7,8^.

### Motivation

The wide variation in policy responses to COVID-19 across countries can largely be attributed to a global lack of experience in dealing with pandemics at this scale. COVID-19 forced many countries to adopt various non-pharmaceutical interventions (NPIs) as community mitigation strategies. NPIs are actions taken, besides the use of medicine and vaccines, to flatten the disease transmission curve^9^. While NPIs have proved to be important in mitigating the spread of the virus and ensuing deaths over time, and across waves^10,11^, certain policies had terrible effects on national economies, from which some nations are still yet to recover^12–14^.

In this paper, we perform a global analysis of various policies rolled out by governments. We study the effect that these policies had on the spread of the virus while taking into account the socioeconomic factors at play. We seek to provide models and evidence for guiding effective government policies in the early (pre-vaccination) stages of a pandemic to yield the highest compliance from citizens without severely compromising the economy and quality of life of people needlessly.

### Objectives

In this paper, we seek to answer the following four research questions.

1. How did COVID-19 (cases and deaths) and stringent policies affect mobility?
2. Which policy measures are most effective for managing COVID-19?
3. What are the influences of socioeconomic and demographic factors?
4. How has compliance changed with the emergence of variants?

### Literature review

COVID-19 has had far-reaching global effects, leading to a worldwide concerted effort to curate relevant data and generate research necessary to understand the virus, responses, and eventual outcomes.

As Berger et al.^15^ note, governments all over the world were faced with the arduous task of making policy decisions under high levels of uncertainty in an attempt to halt the spread of the virus, and when that failed, they had to make more tough decisions on the timing, duration and intensity of interventions to minimize case counts and mortality, primarily through attempts to enforce social distancing and consequently reduce transmission rates^6^. This is commonly referred to as “flattening the curve”, and according to a forecasting study on the United States of America^16^, could have potentially resulted in benefits valued at about $5.2 trillion if implemented effectively.

In the pre-vaccination stage of the pandemic, the policies implemented by governments were NPIs rolled out at country and state levels with different levels of enforcement ^7,11^. One of the policies most advocated for was the use of facial coverings, and primarily, nose masks^17,18^. According to Mitze et al.^19^ not only did face masks considerably reduce COVID-19 cases in Germany, achieving a 45% reduction in a span of 20 days, but they also have negligible economic costs when compared to other public health measures.

With what is hopefully the worst of the pandemic behind us, we, as a planet, are trying to adapt to life with COVID-19; what many have called a “new normal”^20,21^. However, while we have learned to live with COVID-19, experts still warn of the possibility of future pandemics^22,23^. It is imperative that we learn as much as we can about the optimal blend of policies for addressing a pandemic based on our experience with the novel coronavirus during the last two years. It is especially important to understand the efficacy of different policies that are meant to address the effects of the virus in the period before vaccines are available. This allows us to prepare ourselves for the possibility of future pandemics.

Retrospective studies performed at a country level have helped to assess the effectiveness of certain policies against COVID-19. For example, the use of facial coverings has been found to be most effective and cost-effective in Germany^19^. Azman and Luquero^24^ suggest that China’s extreme lockdowns, active case surveillance, and other rapid control measures led to substantial reductions in transmission as of late March 2020, although this was at the expense of the social and emotional well-being of many and has resulted in slower economic growth. In Rwanda, in addition to early action and effective use of social media for information dissemination, robots were employed for patient monitoring in hospitals^25^.

Considering that the operational contexts of every country are different and that certain policies cannot be readily transferred, it becomes necessary to take a holistic look at the policies on a multinational scale to test empirically which ones perform adequately regardless of the country in question. The Organisation for Economic Co-operation and Development (OECD) attempted such an analysis^26^ but was limited to just 18 out of its 38 member states. Their paper acknowledged the role of income support and debt relief as motivating factors for people required to stay at home but raised the issue of poor targeting in terms of who needed this kind of support the most, and whether they had the right access. This is in line with assertions that an understanding of socioeconomic variations in government and citizen responses is required for pandemic governance^27^. The OECD finally concedes the need for extra investigations to make the right policy recommendations for member states. A study of 37 countries examined the effectiveness of policies in response to the first COVID-19 outbreak and found that the greater the strength of government interventions at an early stage, the more effective these are in slowing down or reversing the growth rate of deaths^28^.

### Contribution

In this paper, we move past learnings from single countries and international economic organisations like the OECD to pursue a truly global view of the effects of COVID-19 and the effectiveness of NPI mitigation policies using data available through the Our World In Data (OWID) platform.

Through our investigations, we provide an empirical analysis of the effects of government policies on the spread of the virus, while taking into account the effects of socio-economic factors on the general compliance of citizens, especially with regard to mobility-limiting restrictions, which can have severe economic consequences.

We quantify compliance as the correlation between stringency and relative residential mobility; the relative amount of time people spend at home, as provided by Google mobility trends. Stringency, as used in this paper, refers to policy response combinations adopted by governments to reduce and manage the spread of COVID-19 by limiting contact between citizens. A stringency index, recording the strictness of 'lockdown style' policies that primarily restrict people's behaviour is computed by the Oxford Coronavirus Government Response Tracker (OxCGRT) project^29,30^. We give further details in the data sub-section of our methodology section. Our focus on quantifying compliance is of particular interest to policymakers as failure to effectively change mobility patterns weakens the impact of the intervention.

### Layout

The following subsections detail our methods of analysis, including visualisations and regressions performed to generate insights and explain the contributions of certain features to both case numbers and compliance. We then inspect and draw out insights from our results, followed by data-driven recommendations to serve as a policy guide in the event of future pandemics.

## Methods

### Data and definitions

Through a comprehensive aggregation of datasets relevant to COVID-19 on the open-source Our World in Data (OWID) platform^29^, we had access to COVID-19 case data beginning from 28th January 2020 and government policy responses within the same period^30^. Besides a few clearly indicated exceptions, all data referenced in this paper was obtained from OWID. Published data on cases and deaths attributed to COVID-19 are subject to under-reporting^31^. In particular, reported cases are likely to be a substantial underestimate of the prevalence of the disease in a population given that most people with COVID-19 are asymptomatic, and even among those who are symptomatic, not all are tested. While acknowledging these data limitations, our study focuses on visualising the evolution of the pandemic across several variables, quantifying compliance in terms of reducing mobility and assessing whether or not policies successfully reduced the number of cases.

**Figure 1:**
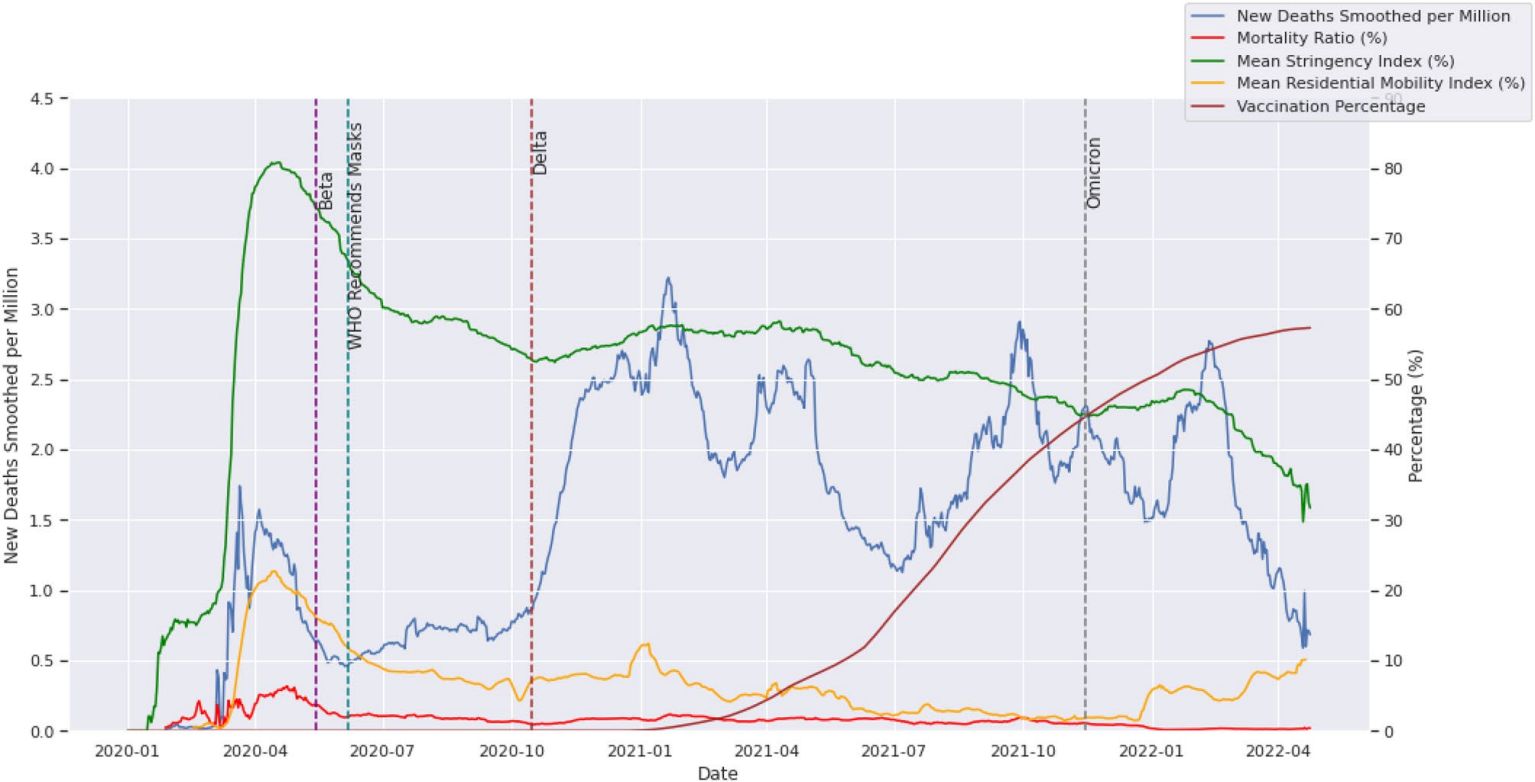
Global evolution of COVID-19 showing the temporal variation in new cases, mortality ratio, mean stringency ratio, mean residential mobility index and vaccination administration.

Our first objective was to assess the effects of COVID-19 on society, policy responses and subsequent changes in mobility. A visual representation is given in Fig. 1. To quantify these effects, we analysed relevant data focusing on five variables of interest.

- New deaths smoothed per million: provides a snapshot of deaths as an intensity metric and how this changes over time across the globe.
- Mortality ratio (derived as the number of deaths smoothed per million divided by the number of cases smoothed per million): measures how deadly the virus is at any point in time, but strictly in relation to the percentage of people confirmed to have died from COVID-19 at that time.
- Stringency index: a composite measure of stringency responses by governments based on the OxCGRT index developed to quantify the extent to which restrictions were applied at a governmental level across the nations of the world.
- Google residential mobility: quantifies the time people spent at home relative to the median value over the five‑week period from January 3rd to February 6th 2020, as captured by Google mobility trends data. This data serves as a measure of the human response to government policies and is our most trustworthy means of empirically determining compliance with mobility restrictions^32,33^.
- Vaccination percentage: a snapshot of the percentage of the global population that, in line with the approach of Hallas et al.^5^, has received at least one dose of any COVID-19 vaccine.

In addition to these variables, we also had to identify when major variants of the virus were first detected. This data was collected from the official WHO records^34^, however, only the month and year were provided so we assumed the 15th day of the given month as the date of first detection. Another important date is the 6th of June 2020, when masks were mandated by WHO^35^.

Our next agenda was to determine the effectiveness of stringency measures implemented by governments. As mentioned earlier, the stringency index is a composite measure, and it aggregates nine different metrics: (1) school closures; (2) workplace closures; (3) cancellation of public events; (4) restrictions on public gatherings; (5) closures of public transport; (6) stay-at-home requirements; (7) public information campaigns; (8) restrictions on internal movements; and (9) international travel controls^29^. All these stringency metrics are primarily meant to reduce mobility and facilitate social distancing. Masks and facial coverings have also been heavily advocated for and therefore data on facial covering policies is included^19^. The impact of these policies over time was defined as the negative correlation coefficient between each policy and the percentage change in the number of cases smoothed per million over a given time period.

Finally, we addressed the issue of stringency policy compliance, defined as the correlation between the composite stringency index and the Google residential mobility index. We investigated how compliance could be explained by socioeconomic and demographic factors, taking the following into account: (1) GDP per capita; (2) Life expectancy; (3) Median age^29^; (4) Literacy rate^36^; (5) Corruption index^37^; and (6) Freedom of expression^38^.

We applied a log transformation to the GDP per capita, which is a standard normalization method to make the distribution more normal. This is also in line with the popular United Nations human development index^39^, which is a combination of the log of GDP per capita, life expectancy and an education index^40^.

Lockdown restrictions are economically costly both to states and to individuals and could have a bearing on how well people comply with directives to stay at home or avoid workplaces. As such, we consider the effects of (1) income support and (2) debt relief policies on the predictability of compliance.

### Analytics

The Pearson correlation coefficient is a statistical standard for measuring the strength of a relationship between two variables. We used it extensively in this paper for calculations of (1) policy compliance, which is assessed using the correlation between stringency indices and Google residential mobility trends, and (2) policy impact, defined as the negative correlation between stringency sub-indices, including policy on facial coverings, and the percentage change in normalised case counts.

Considering that the COVID-19 pandemic has been ongoing for over two years, a certain amount of restriction fatigue^5^ is expected to have developed over time across different countries, which is consistent with the findings of Petherick et al.^41^. Fatigue is visually recognized as a dip in compliance over a period of time. Compliance can be measured and tracked over time by applying a rolling average window to calculate the correlation coefficient between stringency and residential mobility. This window needs to be long enough to provide sufficient data for reliable statistics but short enough to capture changes in behaviour. We experimented with different window sizes and found that three months was most effective for this purpose.

We divided the pandemic window into three major time periods: the uncertainty period (January–March 2020) which represents a break between the normalcy of 2019 and the declaration of COVID-19 as a pandemic by WHO^2^; the pre-vaccination period (April–December 2020); and the vaccination period (January 2021 to the present). The analysis below focuses predominantly on the period from 1st April 2020 through 31st December 2020. This was the period before the first major rollout of vaccinations and within which much of the uncertainty associated with the pandemic was decreasing. Essentially, this pre-vaccine period is the most relevant period within which we can assess the effectiveness of different policies. Once the vaccines were rolled out, there was a marked change in behaviour with many people becoming much less risk-averse than they had been previously. It can be argued that this period is therefore a stable period of time for quantifying policy impact and compliance.

To quantify the average policy impact, I(k), for a period of the following k days, we pooled daily country-level policy index data, x(t), for the pre-vaccination period (1st April through 31st December 2020) and computed the correlation with the relative change, g(t,k), in normalised case counts smoothed per million, y(t), over a period of k days. These calculations were performed on a range of horizons, k, from 0 to 84 days (12 weeks):

1 $${\text{I}}\left( {\text{k}} \right) = - {\text{corr}}\left( {{\text{x}}\left( {\text{t}} \right),{\text{ g}}\left( {{\text{t}},{\text{k}}} \right)} \right)$$

2 $${\text{g}}\left( {{\text{t}},{\text{k}}} \right) = {\text{y}}\left( {{\text{t}} + {\text{k}}} \right)/{\text{y}}\left( {\text{t}} \right) - {1}$$

To assess the influence of socioeconomic and demographic factors on compliance, we ran regressions on a variety of country-level factors within the pre-vaccination period against the average compliance of that country within the same period. We took into account the income support and debt relief indices so as to quantify the effects of these policies on the compliance levels. The results are studied across the globe and on a continent level during the pre-vaccination period. The adjusted R-squared is used as a measure of the goodness of fit for each model.

In order to ensure the robustness of our regression analyses to potential overfitting we calculated the cross-validated R-squared value by leaving out one country in each fold of our cross-validation technique. Leave-one-out essentially provides an out-of-sample result for each country by estimating the model on the remaining N − 1 countries. Hence this is more computationally intensive than the traditional in-sample approach because instead of fitting a single model, it is now necessary to construct a separate model for each country that is left out. All the results are then combined to provide the final cross-validated R-squared result.

## Results

### Effects of COVID-19 and policy responses on mobility

Figure 1 gives a global long-term temporal view of the spread of the virus (blue) and the associated mortality rate (red) as well as the average stringency index (green) and the human response in terms of the Google residential mobility index (orange), indicating the relative change in the amount of time people spend at home, compared to the median value for the 5‑week period from 3rd January to 6th February 2020. Super-imposed on all of this is the percentage of the global population with at least one dose of the vaccine (brown). We also divide the graph into separate sections using specific dates of interest, particularly the day on which WHO recommended mask mandates and the dates on which the major variants were estimated to have first been detected.

The period before April 2020 was marked by a lot of uncertainty about the virus, its transmission, treatment, and prevention, among other things. The first wave of deaths in this period was met by lockdowns and extreme restrictions on mobility, with the global average stringency index reaching a high of 80.83% on 18th April 2020. Just six days after this peak in restrictions, the highest mortality ratio of 6.35% was recorded on 24th April 2020. Eventually, concerted global efforts of restricting human mobility followed by a WHO recommendation for facial coverings (masks) had the desired effect of reducing the mortality rate and keeping it under 2.0%.

After the initial lockdowns, there have been more waves of the virus, and while not synchronized across countries, these have been marked by the emergence of different variants: Beta, Delta, and Omicron (Fig. 1). According to WHO^34^, these variants were estimated to have emerged in May 2020 (Beta), October 2021 (Delta), and November 2021 (Omicron), with Alpha being the originally sequenced variant. Omicron proved to be the most contagious, with cases reaching a record high of over 3.5 million cases in January of 2022.

From Tables 1 and 2 we can accurately track the levels and changes that occurred between key dates for these important variables. For example, during the period from when the virus was declared a pandemic on 11th March 2020 until the peak of the mortality ratio of 6.35% experienced on 24th April 2020, the normalized case count grew by 157.85%. This was within the period of the first global lockdown in which more than 100 countries instituted a full or partial lockdown^42^. The lockdowns led to an 8.21% reduction in the normalised case count and a 2.65% drop in the mortality ratio by the time the Beta variant was detected in May 2020. Throughout this period, compliance was consistently above 80%, indicating that people were actually staying at home and following recommended guidelines.

**Table 1:** Global averages for mortality ratio, stringency index, residential mobility, new cases smoothed per million, and compliance on key dates.

Period/event	Date measured	Mortality ratio (%)	Stringency index (%)	Residential mobility (%)	Mean new cases smoothed per million	Compliance with 3-month window (%)
Pandemic status declared	11/03/2020	1.19	26.31	0.90	6.05	NA
Uncertainty period	24/04/2020	6.35	79.78	20.31	15.60	87.80
Beta detected (South Africa)	15/05/2020	3.70	74.29	15.95	14.32	84.43
WHO recommends masks	06/06/2020	1.99	66.96	11.89	20.17	77.94
Delta detected (India)	15/10/2020	0.92	52.88	7.04	83.51	31.51
Start of vaccine rollout/end of 2020	31/12/2020	1.76	56.69	11.56	129.16	37.44
Omicron detected (multiple countries)	15/11/2021	1.09	45.0	1.83	199.01	24.24
End of 2021	31/12/2021	0.36	46.29	5.46	386.21	21.30
Height of omicron	26/01/2022	0.19	48.47	6.3	1157.70	19.85

**Table 2:** Percent change since previous date in global averages for mortality ratio, stringency index, residential mobility, new cases smoothed per million, and compliance on key dates.

Period/event	Date measured	Change in mortality ratio (%)	Change in stringency index (%)	Change in residential mobility (%)	Mean new cases growth rate (%)	Change in compliance with 3-month window (%)
Pandemic status declared	11/03/2020	NA	NA	NA	NA	NA
Uncertainty period	24/04/2020	5.16	53.47	19.41	157.85	NA
Beta detected (South Africa)	15/05/2020	− 2.65	− 5.49	− 4.36	− 8.21	− 3.84
WHO recommends masks	06/06/2020	− 1.71	− 7.33	− 4.06	40.85	− 7.69
Delta detected (India)	15/10/2020	− 1.07	− 14.08	− 4.85	314.03	− 59.57
Start of vaccine rollout/end of 2020	31/12/2020	0.84	3.81	4.52	54.66	18.82
Omicron detected (multiple countries)	15/11/2021	− 0.67	− 11.69	− 9.73	54.08	− 35.26
End of 2021	31/12/2021	− 0.73	1.29	3.63	94.07	− 12.13
Height of omicron	26/01/2022	− 0.17	2.18	0.84	199.76	− 6.81

On 6th June 2020, WHO officially recommended the use of masks, and in the four months between then and when the Delta variant was first detected in October, the mortality ratio more than halved from 1.99 to 0.92%. Nevertheless, with a drop in compliance from 77.94 to 31.52%, we see a 314% increase in the number of cases in the same period. With the advent of Delta, and moving into the end of 2020 we observe an 18.82% increase in compliance as the mortality ratio climbed back up to 1.76%, suggesting that people’s behaviour, in terms of risk aversion, is largely motivated by the perceived deadliness of the virus at a given time.

In the eleven months following vaccination rollouts, the mortality ratio dropped back down to 1.09% and compliance saw a corresponding drop to 24.24% despite three waves of the Delta variant. At this point, in November 2021, the Omicron variant emerged, and with it, a 94% surge in the normalised case counts by the end of 2021. However, the mortality ratio dropped to 0.36% and compliance dropped to 21.3% in the same period. It is believed that the Omicron variant is more contagious but less deadly than the Delta variant^43^.

Omicron went on to reach an all-time high normalised case count of 1158 new cases smoothed per million on 26th January 2022, marking an almost 200% increase in just 26 days. The mortality ratio, however, dropped to 0.19% in that same period. With the continuous drop in mortality ratio throughout 2021 and going into 2022 we see that even though many people became infected and tested positive, the risk of fatality was successfully managed downwards as time progressed.

With the drop in the mortality ratio, we also see a drop in stringency and a corresponding drop in residential mobility (people staying at home). For example, at the height of the first wave on 24th April 2020, the global average stringency is 79.78% and residential mobility is 20.31%. However, by the last day of 2021 stringency has dropped to 46.29% and residential mobility has declined to 5.46%. The correlation between stringency and residential mobility provides a useful measure of compliance, and the changes in compliance over time demonstrate how human behaviour has varied. The factors influencing compliance are many and of course vary from one person to the next. For example, a UK study^44^ highlighted increased symptoms of fatigue based among males, the divorced, part-time employees, and/or parents of more than two children during periods of warmer temperature. By considering a number of variables, we can, however, offer some insights about how populations respond in aggregate.

**Figure 2:**
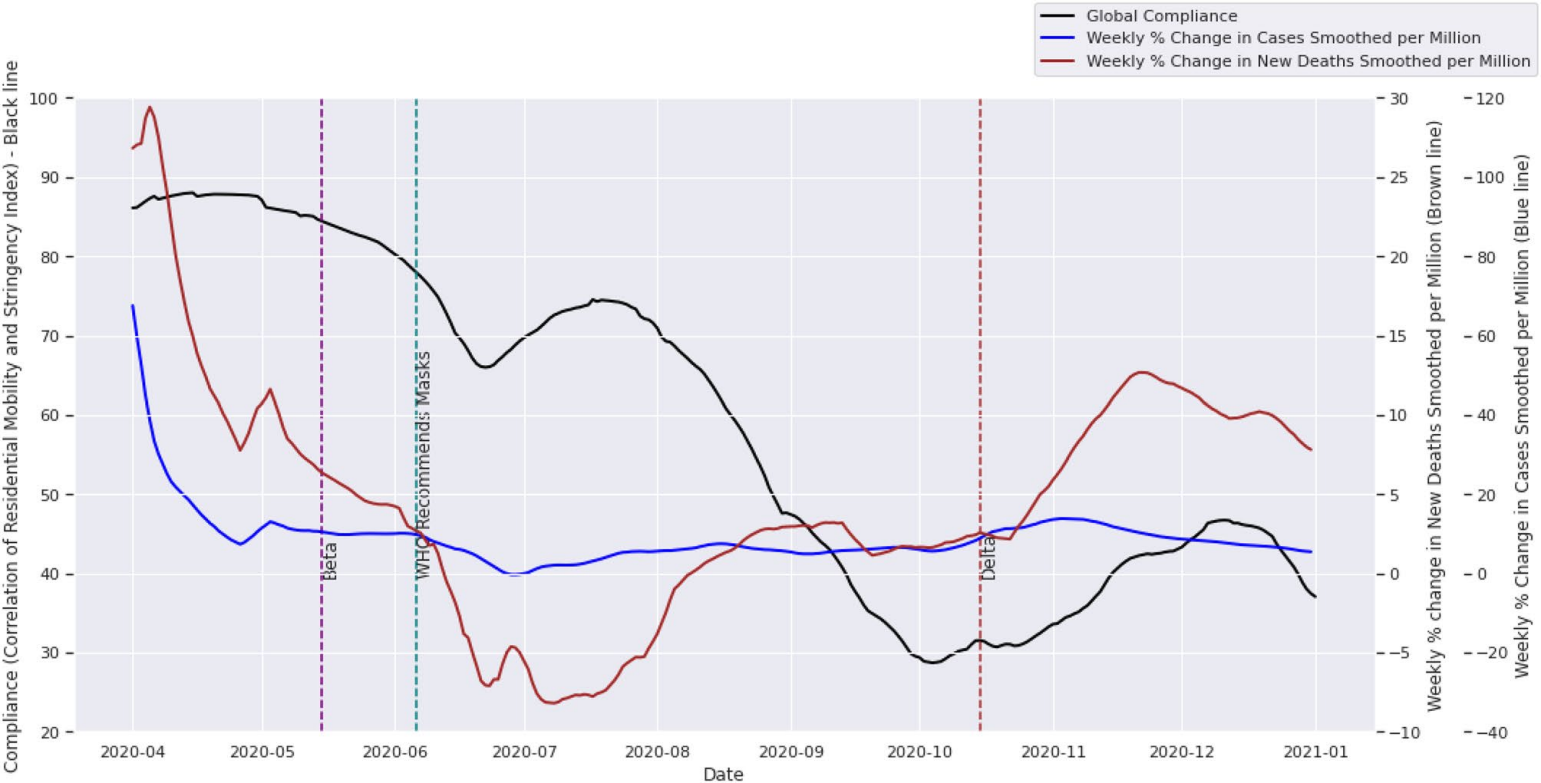
Average compliance levels for the globe using rolling windows of three months from April 2020 to December 2020 (the pre-vaccination period) superimposed on the weekly percentage change in deaths smoothed per million and the weekly percentage change in case counts smoothed per million.

Using a moving three-month window, we plotted the evolution of compliance over the pre-vaccination period of 2020, using a moving average window of three months, comparing the global evolution of compliance to the weekly percentage changes in new deaths smoothed per million and new cases smoothed per million (Fig. 2). The detrimental effect of non-compliance is evident in this graph. As compliance decreased from almost 75% in July 2020 to less than 30% in October 2020, the week-on-week death rate increased from a low of − 8% in July 2020 to eventually peak at 12% in November 2020.

**Figure 3:**
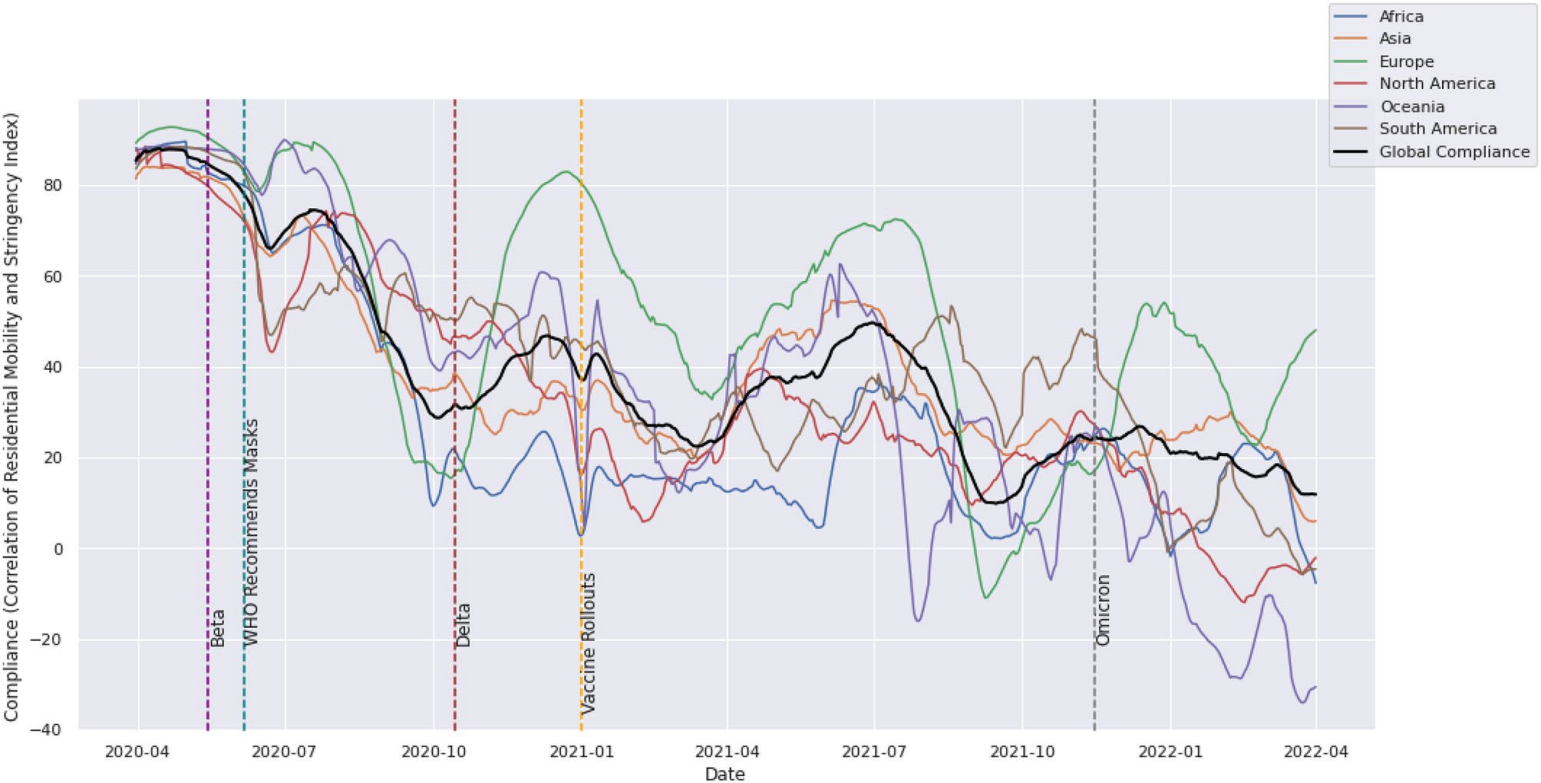
Average compliance levels for the globe and each continent using correlations estimated using rolling windows of three months between April 2020 and April 2022.

A similar plot was made using a three-month window to show the evolution of compliance over the 2-year period of varying restrictions from April 2020 to April 2022 (Fig. 3). The plots were made for countries grouped by continent, with global compliance superimposed.

From Figs. 2 and 3 we see an obvious downward trend in compliance over time; a clear indicator of restriction fatigue. Figure 2 gives us a sense of how compliance is affected by changes in case and death counts over time, and under the influence of new variants. Figure 3 gives us a longer-term sense of the fatigue trends in the various continents throughout the pandemic, with occasional peaks shortly following a sharp dip in compliance or the emergence of a new variant.

Fitting an exponential decay curve to compliance in the pre-vaccination period (April 1, 2020 to December 31, 2020), we can estimate the half-life of compliance. Suppose that c(t) is compliance at time t, then exponential decay with a decay constant ɑ is represented as:

3 $${\text{c}}\left( {\text{t}} \right) = {\text{c}}_{0} {\text{e}}^{{ - \upalpha {\text{t}}}}$$

Fitting our compliance time series to the equation, we estimate a decay constant ɑ of 0.004. The half-life is then given by t = − ln(2)/− ɑ. Our estimates found that during the pre-vaccination period, it takes 173 days, a little less than 6 months, for compliance levels to drop to half of the initial value.

From Fig. 2 we see that global compliance up until the middle of 2020 was above 65 percent with a 3-month window, showing a high level of willingness from most citizens in the early days to follow the rules imposed to restrict mobility and successfully manage the pandemic. However, towards the end of 2020, we see a sharp decline in compliance. The largest dip in compliance culminates in a less than 30% compliance level seen in early October 2020. This dip in compliance was followed by the emergence of the Delta variant, after which we see a slight rise in compliance followed by another dip in December around the holiday period. The year 2020 ended with a compliance level of 37.45%. This aligns well with the findings of Ganslmeier et al.^44^, where compliance is shown to be modulated both by weather and social patterns; compliance dips in the typically hotter months and known periods of socialisation.

From Fig. 3, it can be seen that compliance was highest in early 2020 and the disparity between different continents was small, demonstrating a concerted effort to contain the virus using mobility restrictions in the absence of vaccines. As the different continents experienced different waves of the pandemic we begin to see clearer temporal differences in compliance levels. For example, after the emergence of the Delta variant, Europe generally had the highest peaks in compliance (greater than 70% in two cases), while Africa’s compliance was generally low (within the range of 0–20% in most cases). By 2022, compliance had dropped so far down in Oceania and North America that compliance levels were actually in the negative based on the Google residential mobility data.

**Figure 4:**
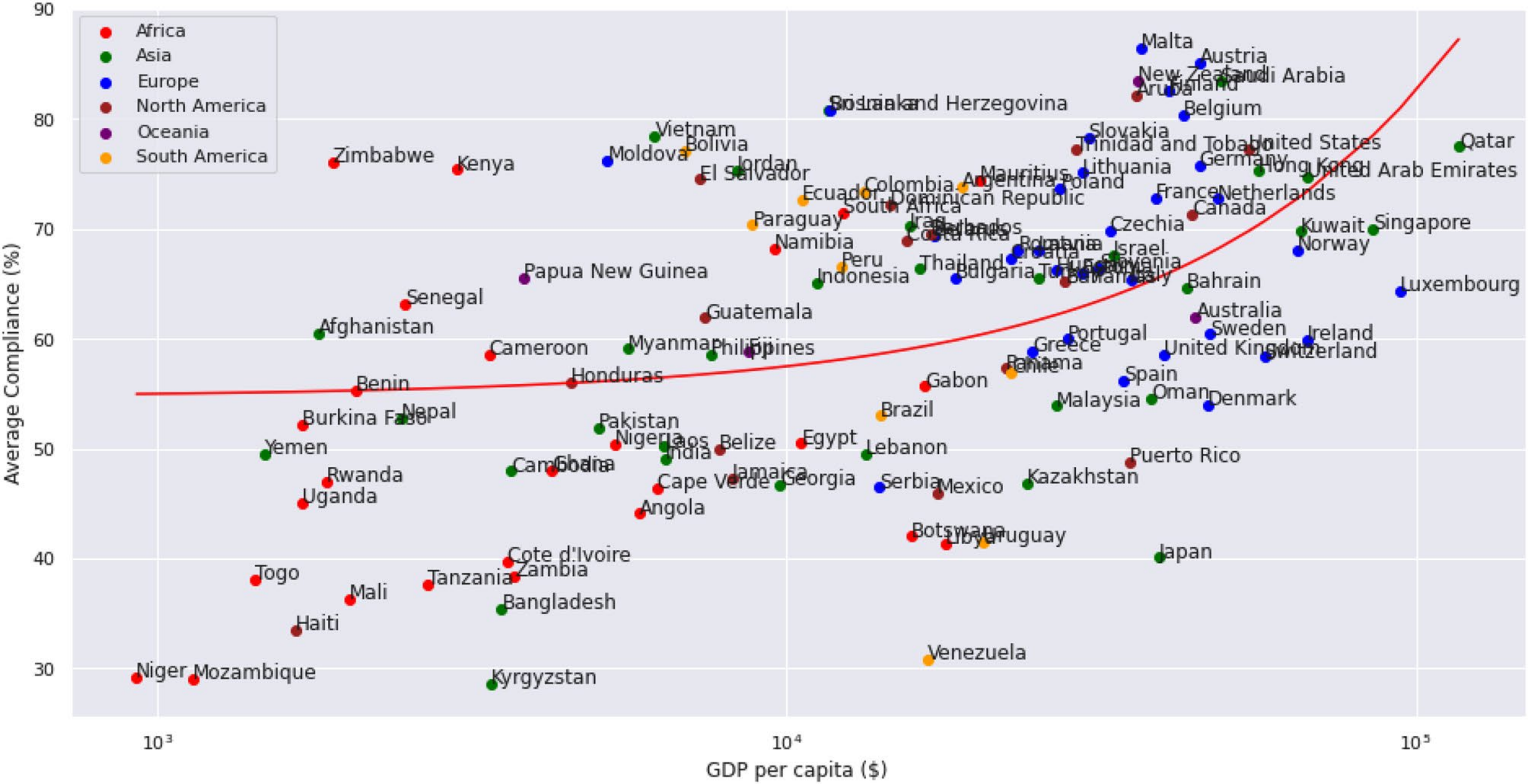
Scatter plot of GDP per capita against compliance for each country in the pre-vaccination period (1st April 2020 through 31st December 2020).

The observations in the pre-vaccine period throughout 2020 are still the most interesting since these are the responses solely affected by NPIs. During this period, the average compliance levels by continent were, from highest to lowest: 68.17%, 67.22%, 61.35%, 59.37%, 54.25%, and 50.52% for the continents of Europe, Oceania, South America, North America, Asia and Africa respectively. At the country level (Fig. 4) we see a trend of increasing compliance with increasing GDP per capita. The nations of South Korea, Nicaragua, Mongolia, and Tajikistan proved to be outliers with average compliance rates of less than 20% and were thus excluded in subsequent regressions to avoid skewing the global analysis.

Major differences are expected across nations based on government support mechanisms that allow people to stay at home. In addition, some countries are more likely to have a larger number of people that already work from home, with many engaged in the digital economy. It is therefore not surprising that wealthier countries can afford to have higher compliance levels, potentially explaining why Europe and Oceania are the most compliant continents.

### Effectiveness of policy measures

**Figure 5:**
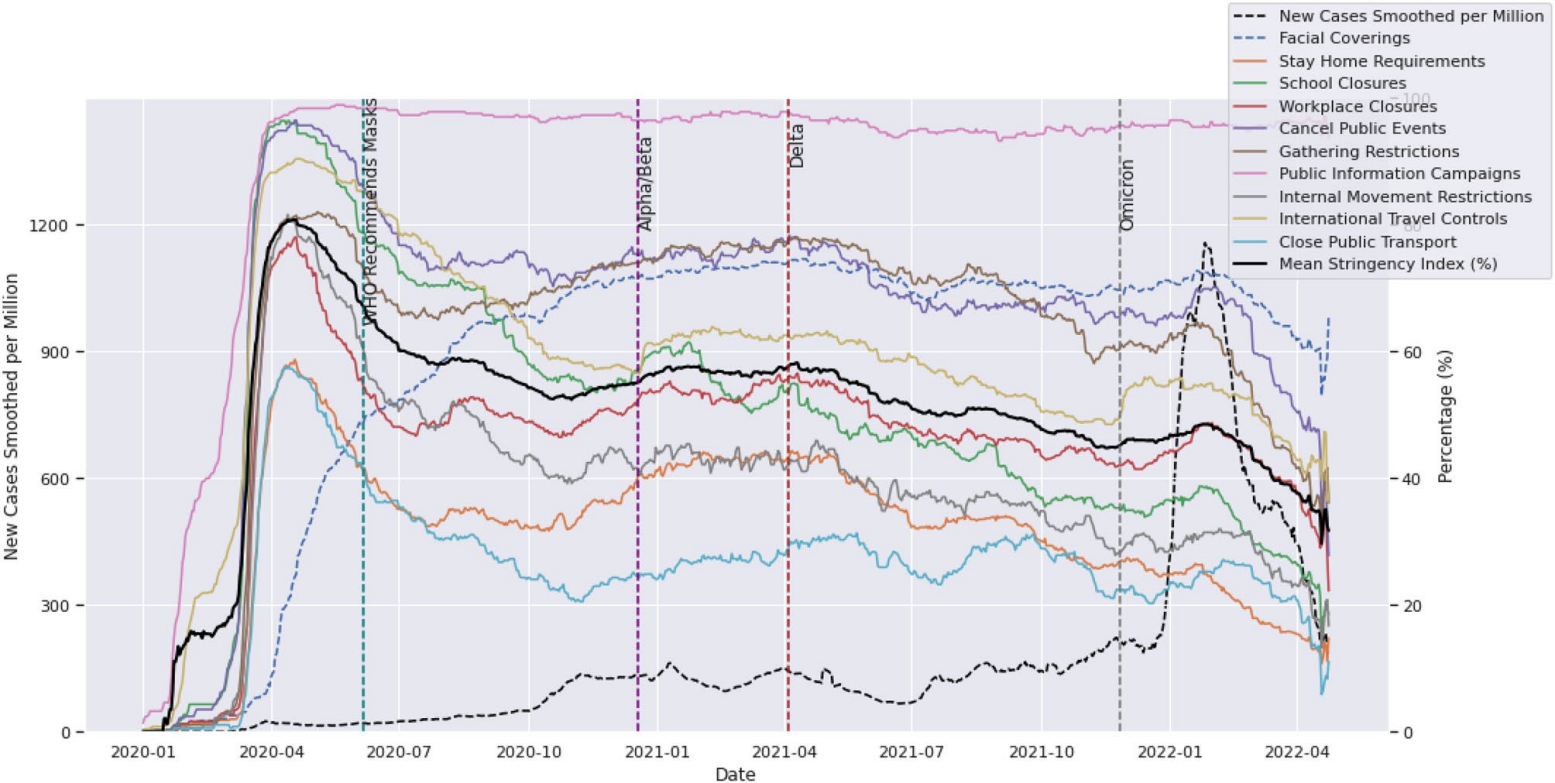
Global temporal variation in mean stringency sub-index, including facial covering index (right), and number of new cases smoothed per million (left).

With the exception of public information campaigns which maintain a high level from April 2020 to the present, the disaggregated stringency measures (Fig. 5) all follow a similar trend, with a spike between March and April 2020 and a slow consistent descent over time. In contrast, policies on facial coverings took off with a much slower start but eventually remained relatively constant around the 70% mark towards the end of the year 2020.

Case numbers generally continued to increase, but the Omicron variant, known to be extremely transmissible, introduced a sharp spike, starting in December 2021 and carried through to April 2022. Fortunately, there was no commensurate spike in the number of deaths in the early part of 2022.

**Figure 6:**
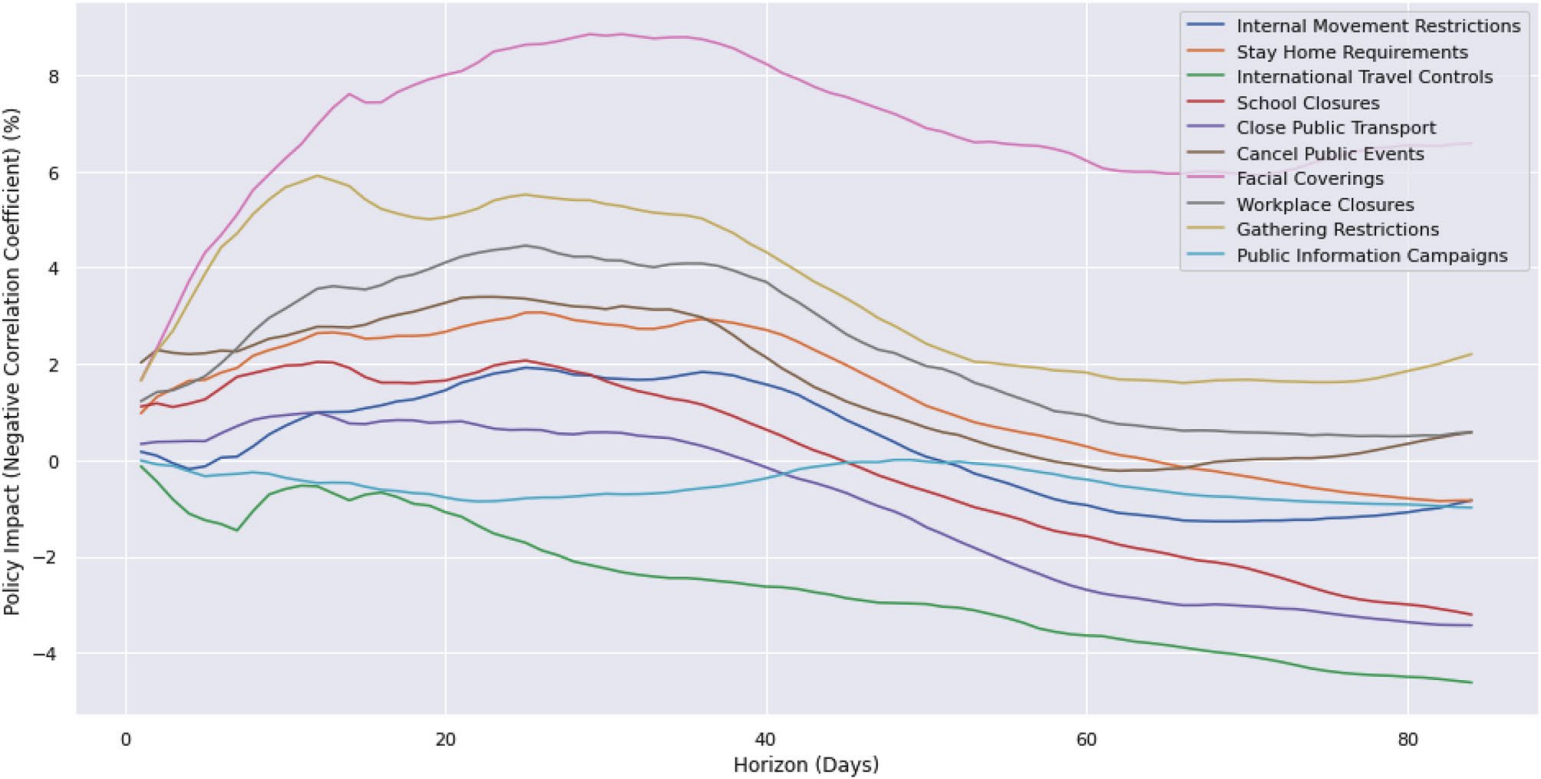
Policy impact quantified using the correlation between government policies and relative changes in normalised case counts for various horizons.

The impact analysis of the government NPI policies investigated how different responses achieve the desired outcome of lowering cases over different time horizons (Fig. 6). For each policy, the maximum impact and corresponding horizon were identified (Table 3). The aim is to identify policies that reduce cases and therefore have a negative correlation with a large absolute value.

**Table 3:** COVID-19 policy responses, impact, and horizon over which policies attain maximum efficiency.

Policy	Impact (%)	Horizon (days)
Facial coverings	8.8	31
Gathering restrictions	5.9	12
Workplace closures	4.5	25
Cancellation of public events	3.4	23
Stay home requirements	3.1	26
School closures	2.1	25
Internal movement restrictions	1.9	25
Closure of public transport	1.0	12
Public information campaigns	0.0	49
International travel controls	− 0.1	1

Facial coverings have the greatest impact by successfully driving down the percentage of new COVID-19 case counts with an optimal horizon of 31 days. With the exception of public information campaigns and international travel controls, all the other stringency sub-indices have a positive impact on the percentage change in the number of cases smoothed per million with their optimal horizons in the range of 12–31 days.

### Influence of socioeconomic and demographic factors on compliance

**Figure 7:**
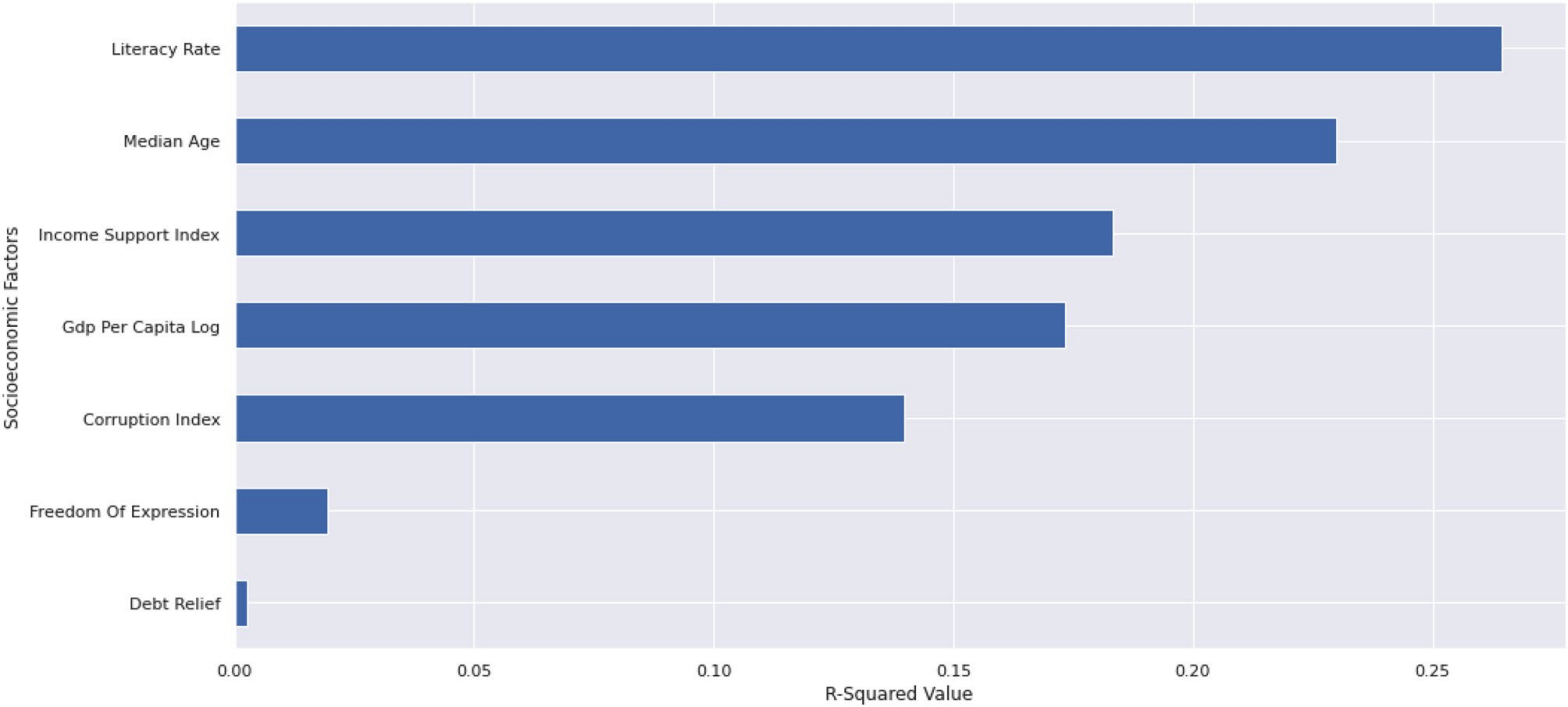
Feature importance of demographic and socioeconomic factors based on the square of correlation coefficients (R-squared) against compliance.

Country-level socioeconomic and demographic factors were used to investigate the variability in compliance across countries. Compliance was positively correlated with all the considered factors, which intuitively makes sense as an increase in any of the factors should result in better compliance. Note that the corruption index is an inverted feature by definition, and a higher corruption index is indicative of less perceived corruption in a country. The square of the coefficients revealed the following order of feature importance (Fig. 7).

Literacy Rate is the most highly correlated variable with compliance. This demonstrates that the presence of a highly literate population is a major factor in determining how willing people are to stay at home during lockdowns. Older populations, the ability of a government to provide income support, wealthier populations, and countries with lower corruption, are also more likely to be compliant with restriction measures.

Co-linearity is an obvious problem with the features highlighted in Fig. 7, so after identifying a model with backward stepwise regression, Literacy Rate and the Income Support index were selected as statistically significant features (p < 0.001 and p < 0.05 respectively). The model had an adjusted R-squared value of 0.293 (Table 4).

**Table 4:** Summary for regression of compliance on country-level features.

Feature	Estimate	T-statistics	P-value
Constant	27.9321	4.983	< 0.001
Literacy rate	0.3213	4.471	< 0.001
Income support index	4.7643	2.581	< 0.05
R-squared	0.305		
Adjusted R-squared	0.293		
Cross-validated R-squared	0.262		

While it is clear that income support is effective in driving compliance to stay-at-home requirements, it is also true that many countries do not have the means to implement this. According to a World Bank report^45^ ninety percent of countries reported a decline in GDP per capita in 2020 and an estimated 120 million people were pushed into extreme poverty. The richer countries of the world would need to help developing economies in order to use income support as a means of improving policy compliance in the face of contagious disease outbreaks.

An increase of 1% in the literacy rate of a country is associated with a 0.32% increase in compliance (Table 4), meaning that a country with an 80% literacy rate is likely to see 3.2% more compliance than one with a 70% literacy rate. This is in line with Rodon et al.^46^ who assert that people with a higher COVID-19 health literacy adopt more protective behaviours.

Income support index, on the other hand, takes on the values 0, 1 or 2 depending, respectively, on whether zero financial support is available, up to half, or greater than half of one’s previously earned income was provided by the government. This regression suggests that providing income support of more than half of previous earnings is associated with an increase of 4.76% in the compliance level (Table 4).

**Figure 8:**
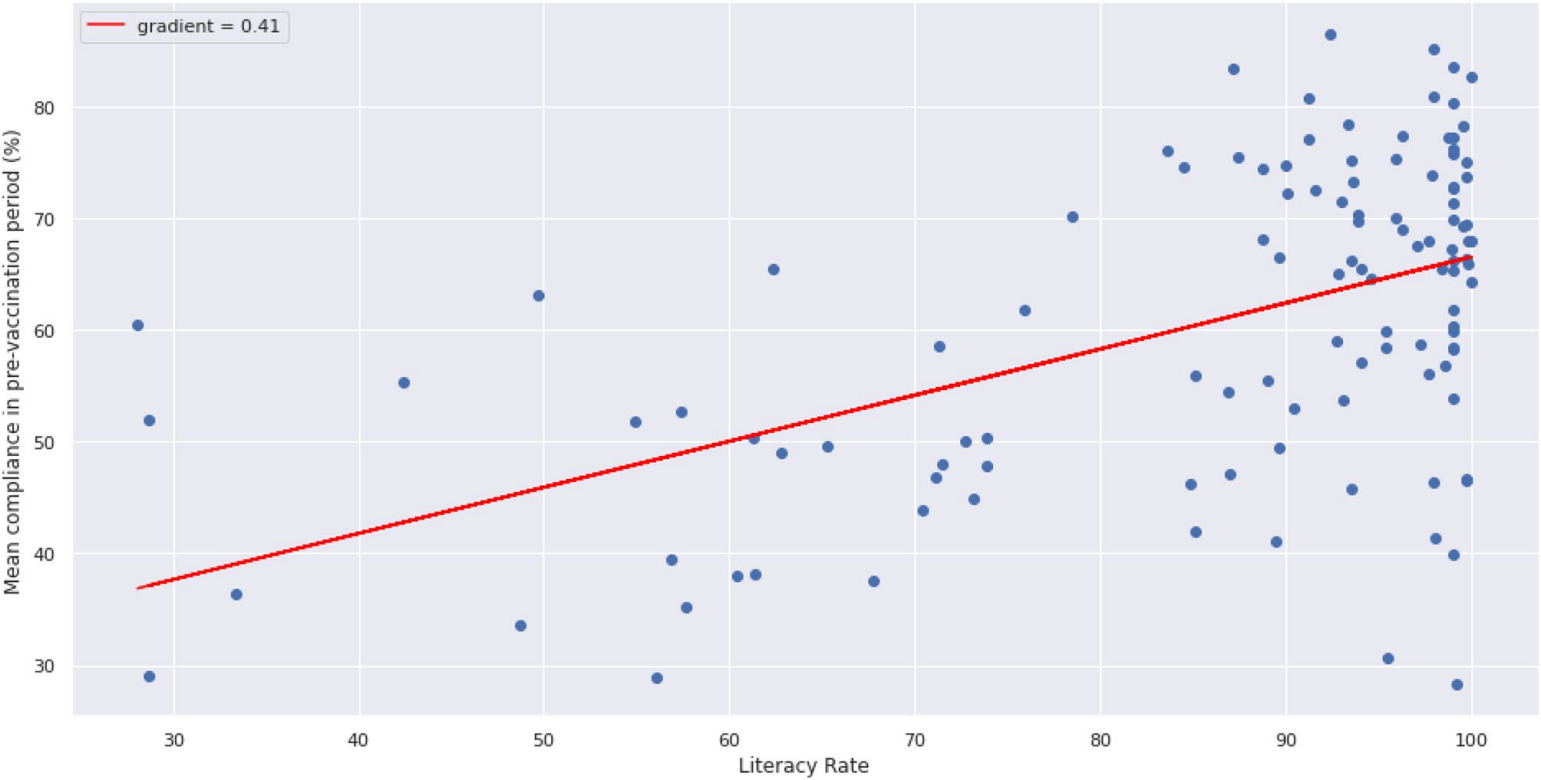
Linear regression model using the literacy rate as the selected independent variable and compliance as the dependent variable.

**Figure 9:**
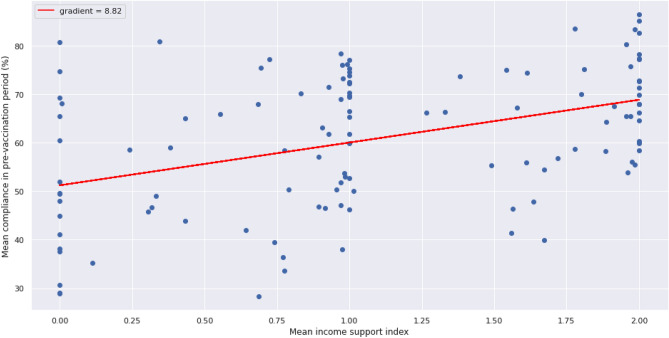
Linear regression model using income support as the selected independent variable and compliance as the dependent variable.

Univariate linear regressions on the selected features estimate even more gains in compliance for each feature with an estimated 0.41% increase in compliance per unit percentage increase in literacy rate (Fig. 8) and an estimated 8.82% increase in compliance per unit percentage increase in income support (Fig. 9).

## Discussion

### Effects of COVID-19 and policy responses on mobility

Compliance was defined as the correlation between the stringency index and residential mobility (time spent in residential locations). The high positive values of compliance found in the first half of 2020, averaging over a three-month window (Fig. 2), is proof that people are generally compliant with COVID-19 policies, but fatigue tends to set in once the number of deaths begins to decline (Fig. 2). In the first half of 2020, global citizens maintained a compliance level higher than 65% on average. Towards the end of the first year of the pandemic, however, there was a significant drop in compliance across the entire globe, which is evidence of further fatigue. Vaccinations have not solved the problem of increasing COVID-19 case counts, but have been strongly associated with the falling mortality rate^47,48^. Lower mortality rates offer people the confidence to resume life as normal, making vaccines a likely motivator for people’s return to a semblance of normalcy. Nevertheless, vaccines are not the only plausible reason for further drops in compliance after 2020.

Economies are sustained by the working population, and eventually, countries had to reopen workplaces to keep their economies afloat. The effectiveness of policies aimed at reducing mobility was studied by regressing compliance on country-level variables. Literacy Rate was the most highly correlated variable that explained when the citizens of a country were able to maintain a high level of compliance. It can be inferred that with a high COVID-19 health literacy rate in a population, people tend to adopt better protective measures. The second most significant variable is the income support index. To ensure that citizens have enough resources to stay at home, income support is clearly a recommended mechanism, since citizens are more likely to comply with stay-at-home directives if they have an alternative means of making ends meet. Median age and the log of GDP per capita are also well-featured values, supporting the assertions that older populations are more compliant due to their vulnerability^49^, and that countries need to have adequate wealth in order to offer resources to sustain workers and families at home.

### Effectiveness of policy measures

It currently takes at least a year to fully develop and test a vaccine to meet international standards^50^. As such, in the event of any new pandemic, policies implemented in the pre-vaccination period are critical for suppressing the spread of infections. Determining how citizens respond to different policies until a more permanent solution can be developed and applied at a national or global scale is therefore of great importance.

There are two major considerations for such policies: the first is the effectiveness of the policy in controlling the spread of the disease, and the second is the effect of the policy on the livelihoods of people. Any extended negative impact on the livelihoods of people will weaken the effectiveness of a policy due to fatigue and eventually a lack of compliance.

Our study using information about the stringency of a particular policy and the number of cases for each day and country allowed for the quantification of the impact over different temporal horizons. It provided a comparison of the impact of various policies and the horizon over which they take maximum effect. It is evident that most policies require well over 20 days to yield any effect, and that the impact is marginal for some of these policies. It is also worth noting that it takes at least 12 days for any single policy to effectively contribute to a decline in case counts as seen in the case of Gathering Restrictions and Closure of Public Transport (Fig. 6).

Facial coverings have the highest impact at 8.8% and play an important role in reducing the number of COVID-19 cases within a period of approximately one month, while also being the most cost-effective method, as confirmed by Mitze et al.^19^. Gathering restrictions are the most useful for achieving a short-term impact of 5.9%. Workplace Closures, Cancellation of Public Events, Stay Home Requirements, School Closures and Internal Movement Restrictions all operate over a horizon of around 25 days and had decreasing impacts of 4.5%, 3.4%, 3.1%, 2.1% and 1.9% respectively. Closure of Public Transport was also short-term at 12 days but delivered a small impact of 1.0%. Both public information campaigns and international travel controls are found to deliver negligible impact and are therefore difficult to justify based on the global evidence from this study. It is therefore recommended that facial coverings are introduced immediately when a new airborne pandemic emerges as this is both an effective and relatively cheap policy with no adverse effects on mobility or economic growth. School closures had a relatively small impact of 2.1% and it is recommended to focus on the most impactful policies before resorting to this restriction which can have serious long-term effects on the education of children. For example, school closures in Uganda lasted for 82 weeks^45^ adversely affecting many students. The minor impact of school closures was previously echoed in the findings of the heavily cited study by Viner et al. ^51^ which concluded that “school closures alone would prevent only 2–4% of deaths, much less than other social distancing interventions”.

### Influence of socioeconomic and demographic factors on compliance

The greatest influence on compliance is the literacy rate, followed by income support. Both variables are selected in backward stepwise regression at a significance level of p < 0.05 (p < 0.001 for literacy rate), with an adjusted R-squared value of 29.3%. This finding suggests that these variables are statistically significant in explaining the compliant behaviour of those citizens that stayed at home under tight stringency measures.

A cross-validation evaluation was used to test for overfitting and establish the robustness of the results. The objective here is to train and test on different samples, thereby ensuring that the evaluation is fully out-of-sample. This was achieved using leave-one-out cross-validation, whereby one country is left out in each regression fold. This approach yielded a cross-validated R-squared value of 26.2%. As this value is close to our initial adjusted R-squared value of 29.3% we can safely conclude that overfitting is not an issue and therefore confirm a high level of confidence in our analyses.

## Conclusions

In this paper we have empirically analysed the effects of COVID-19 case counts and deaths on mobility. We observed how increased numbers of deaths increased the mortality ratio in the pre-vaccination period leading to lockdowns and other high stringency measures, causing people to stay at home in line with policies. We also observed the effects of policy fatigue as compliance waned with every reduction in the number of deaths.

With the variants of concern observed in this paper, we see a trend where a new variant leads to a spike either in deaths or in cases (the former being more seriously impactful). With this increase comes more reactive stringency measures from governments, leading to compliance for as long as the wave lasts. By the end of each wave, measures are relaxed and life returns to some semblance of normalcy. With each new variant, however, people appear to have become less risk-averse, especially with the downward trends of the mortality ratio and the successful administration of vaccinations. Effectively, based on a multiplicity of factors, compliance has reduced despite the emergence of new variants.

We came to the conclusion, in line with existing research^17,18^, that face coverings are the most effective intervention as well as the most cost-effective, associated with the highest reductions by percentage in the number of COVID-19 cases after approximately a month.

### Recommendations

Based on the relative efficacy of masks, we recommend the mandatory use of face masks as the best first step in the pre-vaccination stage of any airborne disease. Gathering restrictions are also advisable in the short term, achieving maximum impact over a short period of just 12 days. Workplace closures, cancellation of public events and stay home requirements are also advisable in countries with a large digital economy. Where there is the ability to work remotely and organize events online without having to leave home, there is an opportunity to curb the effects of the pandemic without delivering a crippling shock to the economy. In countries with low levels of digitization and poor internet penetration, the options may be limited and it is necessary to carefully balance public health with economic considerations. There already exists evidence that COVID-19 has increased global income inequality, partly undoing two decades of progress in lowering inequality and disproportionately affecting vulnerable groups and Emerging Markets and Developing Economies (EMDEs), where income inequality is considerably higher than in advanced economies^52^.

We do not advise school closures except as a last resort because of the terrible implications for students and their future^53^, with further evidence in the case of Uganda^45^, and the particularly harsh effects school closure has on girl-child education^54^. Restrictions on internal motion and public transport are likely to have an effect on the economy because of how critical mobility is to business. Public information campaigns have been so consistent since the start of the pandemic that they no longer offer any explainability. International travel restrictions are only useful in the very early days of a pandemic, but our analysis suggests that once cases are recorded internally in several countries, they become unnecessary as a mitigation measure.

Policies for managing a pandemic are only as effective as the citizens are cooperative, meaning that governments should put measures in place to keep fatigue at a minimum and avoid risking the livelihoods of citizens for extended periods of time. Income support and GDP per capita are strong indicators of likely compliance. Essentially, the financial standing of a nation explains its ability to keep citizens comfortable enough to abide with restrictions. If income support is not an option based on available finances, lockdowns will likely fail, and it is more advisable for poorer nations to enforce the use of masks and implement other social distancing measures.

### Limitations

The data available only permitted us to calculate compliance based on mobility. While we know what policies are being rolled out by governments we have no way of knowing how people comply with non-mobility related policies, like the wearing of face coverings.

Another limitation was accurate information about variants. The information on emergence of variants often lacked temporal accuracy due to few countries having the required technical capacity to detect variants. This implies that reasonable assumptions have to be made about when variants were actually first discovered. There is also a lack of information on the percentages of variants circulating at any point in time, forcing us to disregard the possibility that certain previous variants might still be circulating with significant impact.

We also encountered a lack of data on the monetary valuation of policy implementation costs. A lot of the existing analysis focuses on the social and wellness costs of policies. Unfortunately, without any explicit financial data on the spend for policy implementations, there is no practical way of undertaking an extensive cost–benefit analysis across the globe to understand which policies make the most financial sense, and what the actual monetary cost is when implementing such a policy.

## Data Availability

The datasets analysed during the current study are available in the Oxford COVID-19 government response tracker (OxCGRT) repository, https://ourworldindata.org/coronavirus. The Google Mobility Trends datasets can be accessed from Google’s COVID-19 Community Mobility Reports, https://www.google.com/covid19/mobility/. Finally, the data used to estimate the emergence of COVID-19 variants was found on the Word Health Organization’s official web page, https://www.who.int/en/activities/tracking-SARS-CoV-2-variants/.
